# Virtual reality in psychological interventions for mood disorders: a scoping review

**DOI:** 10.1186/s12888-026-08088-9

**Published:** 2026-04-21

**Authors:** Yufei Wang, Kehua Yang, Tingting Yang, Ping Xu, Miaomiao Lin, Yujing Fan, Yuexian Tao

**Affiliations:** 1 School of Public Health and Nursing, Hangzhou Normal University, Hangzhou, Zhejiang China; 2 Sir Run Run Shaw Hospital, School of Medicine, Zhejiang University, Hangzhou, Zhejiang China

**Keywords:** Virtual reality, Psychological interventions, Mood disorders, Depression, Bipolar disorder, Scoping review

## Abstract

**Background:**

Mood disorders impose substantial physical and psychological burdens globally. Virtual reality (VR)- based psychological interventions have gained attention as a potential approach in this field. However, current research on these interventions for mood disorders remains heterogeneous and lacks a comprehensive synthesis. A scoping review is needed to map the existing evidence, and to identify trends and gaps in this emerging area.

**Objective:**

This scoping review was to synthesize the existing status of applications about virtual reality-based psychological interventions for mood disorders by examining the characteristics and reported outcomes of included studies.

**Methods:**

This scoping review was conducted following Arksey and O’Malley’s methodological framework. We performed systematic searches across seven electronic databases and gray literature sources. The search encompassed all available records from database inception to March 2026, focusing on studies that were about VR-based psychological interventions for mood disorders.

**Results:**

18 studies were included, finding four types of VR-based psychological interventions, namely cognitive-oriented, experiential-oriented, social situation simulation, and psychoeducational tools. Based on user interaction modes, these interventions were classified into immersive interactive and non-interactive categories. Interventions generally included 1–24 sessions (4–90 min per session, 1–2 sessions weekly), with the most common being 4–12 sessions of 30–50 min each. All studies assessed efficacy, with depressive symptoms and emotion regulation as the most frequent outcomes. 55.6% included feasibility evaluation. User experience feedback highlighted five themes, including interaction design, environmental fidelity, hardware suitability, user experience, and reality transferability.

**Conclusions:**

This review identified four intervention types by content and therapeutic orientation and two categories by user interaction modes. VR-based psychological interventions for mood disorders remain at an early stage. Future research should develop standardized intervention frameworks and conduct more high-quality trials across diverse populations to confirm their effectiveness.

**Clinical trial registration:**

Not applicable.

**Supplementary Information:**

The online version contains supplementary material available at 10.1186/s12888-026-08088-9.

## Introduction

Mood disorders (MD) are mental disorders characterized by persistent and significant affective dysregulation. According to the DSM-5 diagnostic framework, mood disorders are divided into specific categories, including depressive disorders and bipolar and related disorders [1]. This review focuses on the most common and typical conditions in this spectrum: major depressive disorder (MDD) and bipolar disorder (BD). Epidemiological data from the Global Burden of Disease Study (1990–2017) indicate a 49.86% global prevalence surge in MDD cases, rising from 172 million to 258 million affected individuals [2]. In 2023 and 2024, the World Health Organization (WHO) reported that there are about 280 million people worldwide who suffer from MDD, and an additional 40 million people have BD [3, 4].

People with MD suffer from impaired psychological functioning, with symptoms varying by diagnosis. MDD is characterized by persistent depressive mood and anhedonia, along with symptoms like hopelessness, guilt, cognitive slowing, fatigue, and suicidal ideation in severe cases [5]. BD is distinguished by two main types of symptoms. Manic or hypomanic episodes involve elevated or irritable mood, increased energy, impulsivity, and decreased need for sleep [6, 7]. Depressive episodes largely resemble MDD. These profound disturbances across both disorders significantly reduce patients’ sense of well-being and life satisfaction. An investigation by Jerome et al. [8] revealed significantly diminished quality of life across all domains among affected individuals compared to the general population. Furthermore, MD is often comorbid with other diseases. Patients may suffer from anxiety, sleep disorders, eating disorders, and other problems [9–11], which are associated with a significantly lower life expectancy [12].

MD causes marked disability, adversely generating considerable burdens to family and society [13, 14]. Between 2007 and 2017, United States healthcare costs attributable to MD rose progressively, showing a $172 billion yearly increase in direct medical expenditures [15]. While MD has a significant economic impact, it has different characteristics depending on the situation. A WHO report estimated that MDD cost €136.3 billion in Europe during 2007 [16]. And its costs stem from direct medical expenditures (e.g., pharmacotherapy) and substantial indirect losses due to impaired work productivity [17, 18]. As for BD, the Global Burden of Disease Study estimated its global impact at approximately 9.9 million disability-adjusted life years (DALYs) in 2013 [19]. According to American statistics from 2015, BD costs the economy $202 billion annually, with indirect costs like lost productivity and unemployment making up 72% of the total [20]. Evidence has recently shown that the incidence of BD has been steadily increasing [21].

The current guidelines state that treatment for MD varies by subtype. For MDD, Cognitive Behavioral Therapy (CBT) and Interpersonal Therapy (IPT) are first-line treatments during acute phases and are still recommended for maintenance [22]; lifestyle and psychological therapies are prioritized for less severe cases, while antidepressant medication combined with CBT or IPT is used for more severe or treatment-refractory cases [22–24]. Pharmacotherapy is the foundation of BD treatment, while psychosocial therapies such as psychoeducation, interpersonal, and social rhythm therapy give additional support throughout the acute depression and maintenance stages to prevent recurrence [25, 26]. Acute mania is managed with medication adjustments, stimulation reduction, and referral to a professional [27]. However, current treatments also have some limitations. Pharmacological treatments exhibit poor tolerability in certain individuals and are associated with cardiovascular complications, metabolic disturbances, and hepatorenal toxicity [28–31]. Although traditional psychotherapies have shown significant efficacy in modifying adverse cognitive patterns, promoting self-acceptance, and enhancing social perceptions [32], their clinical application faces several obvious limitations about therapy accessibility. These include restricted control over the therapeutic environment, limited flexibility in scheduling, constraints in location adaptability, and a shortage of available therapists.

Virtual reality (VR) is an emerging digital intervention technology. It creates virtual environments through multi-sensory stimulation, producing a sense of presence and psychological immersion [33, 34]. VR includes various technical forms, such as fully immersive head-mounted displays (HMDs) and non-immersive screen-based systems [35]. These technologies generate 2D or 3D virtual environments that may include augmented or interactive components [36, 37]. Modern VR systems combine HMDs, data gloves, and haptic suits to simulate human sensory experience and support interaction within virtual settings [38]. This review focuses on VR interventions using immersive HMD technology.

In recent years, VR has been increasingly integrated with traditional psychological interventions for mental health treatment [39–41]. Advances in digital health also create opportunities for treating MD. VR is being studied as a tool to address the time and space limitations of conventional psychotherapy. However, high-quality evidence is still lacking. Therefore, VR is not yet included in clinical practice guidelines and remains experimental [42–44].

Previous reviews have focused on single disorders such as anxiety disorders, phobias, and post-traumatic stress disorder [42]. Clinical trials for depression or bipolar disorder remain in early stages. Existing reviews also do not fully examine VR-based interventions for MD across different technical formats. This scoping review aims to map the current use of VR-based psychological interventions for mood disorders. It also aims to describe the technologies used, identify research gaps, and inform future clinical practice.

## Methods

The study followed the scoping review framework proposed by Arksey and O’Malley [45], covering the following five stages: (1) defining the research question; (2) screening relevant studies; (3) making study selections; (4) graphing the data; and (5) organizing, summarizing, and reporting the results.

In addition, we adopted the PRISMA-ScR checklist (Supplementary Appendix 1) to ensure the quality of the study. The protocol of the scoping review program has been published in the Open Science Framework Registries (registration DOI: 10.17605/OSF.IO/7DSQE).

### Defining the research question

Guided by Arksey & O’Malley’s framework, this scoping review aims to address the following questions:

1. What are the publication trends of relevant studies?
2. What VR-based psychological interventions have been implemented in MDD and BD clinical practice?
3. How are these interventions implemented?
4. What are the effects of these interventions?

### Identification of relevant studies

The study systematically searched the following English databases: PubMed, Web of Science, Embase, CINAHL, Cochrane Library, PsyInfo, and Scopus and gray literature. The search timeframe was from database inception to March 2026. Databases were searched using search terms extracted from MeSH and entry terms, such as “mood disorders” or “bipolar disorder” or “depressive disorder” and “virtual reality” or “VR” or “immersive technology” and “psychotherapy” or “psychological intervention” in the title or abstract of records. The complete search formulas for each database are placed in the Supplementary Appendix 2.

### Study selection

We followed predetermined inclusion and exclusion criteria to guide the selection of this scoping review. Inclusion criteria were as follows: (1) Patients with a definite diagnosis of MDD or BD, confirmed by one of the following: (1) DSM-5 or DSM-IV; (2) the Mini International Neuropsychiatric Interview (MINI); (3) clinical diagnosis by an attending psychiatrist; Mixed-population studies were eligible for inclusion only if MDD or BD were explicitly reported as the primary patient cohort, or their proportional representation within the study sample was clearly specified; (2) The study topic was VR-based psychological interventions for MDD and BD; (3) The type of the study was an original study, including randomized controlled trials, class-experimental studies, case-control studies, mixed studies, qualitative studies, and registered protocols with full intervention details; (4) Studies in the English language.

Exclusion criteria: (1) Non-English study; (2) Duplicate publications; (3) Conference papers; (4) Studies in which VR-based psychological interventions combined with other measures resulting in unmeasurable effects, or studies lacking VR technology application, or not targeting psychological interventions for MDD or BD.

After eliminating duplicate literature, two authors (FYW & TTY) independently read the titles and abstracts and performed the initial screening based on the pre-established inclusion and exclusion criteria. Among them, FYW as the main coordinator, was responsible for summarizing the screening results and organizing the discussion; the other author (TTY), who had experience in evidence-based medical research, assessed the controversial literature. In case of disagreement between the two, a third author (YT) arbitrated and finally reached a consensus. Literature was independently screened in full text by two authors (FYW & TTY), and disagreements were resolved by negotiation. All processes followed the PRISMA-ScR specifications to ensure transparency [46].

### Charting the data

Our research team developed a structured data extraction table, with the main content covering the basic characteristics of the literature (author, country, year of publication, study design, sample size, study population, age), VR-based psychological intervention program (equipment, intervention content, intervention cycle/frequency/duration), outcome indicators and evaluation of the application effect. All data were independently extracted and cross-checked by two authors (FYW & PX) to ensure accuracy. Disagreements were negotiated and eliminated. A third author (YT) retrospectively examined all data.

### Organize, summarize, and report results

The research team systematically discussed and cross-validated all extracted data to ensure their accuracy and consistency with the research objectives. This study used a combination of descriptive statistics and thematic analysis methods to systematically sort out and integrate the characteristics and application effects of VR-based psychological intervention programs. Two methods of data analysis were used in this study: (1) descriptive analysis, which included the number of studies, publication trends and distributions, and calculation of frequencies/percentages for each dimension; (2) thematic analysis, which brought together qualitative data to construct descriptive themes.

## Results

### Study selection

**Fig. 1:**
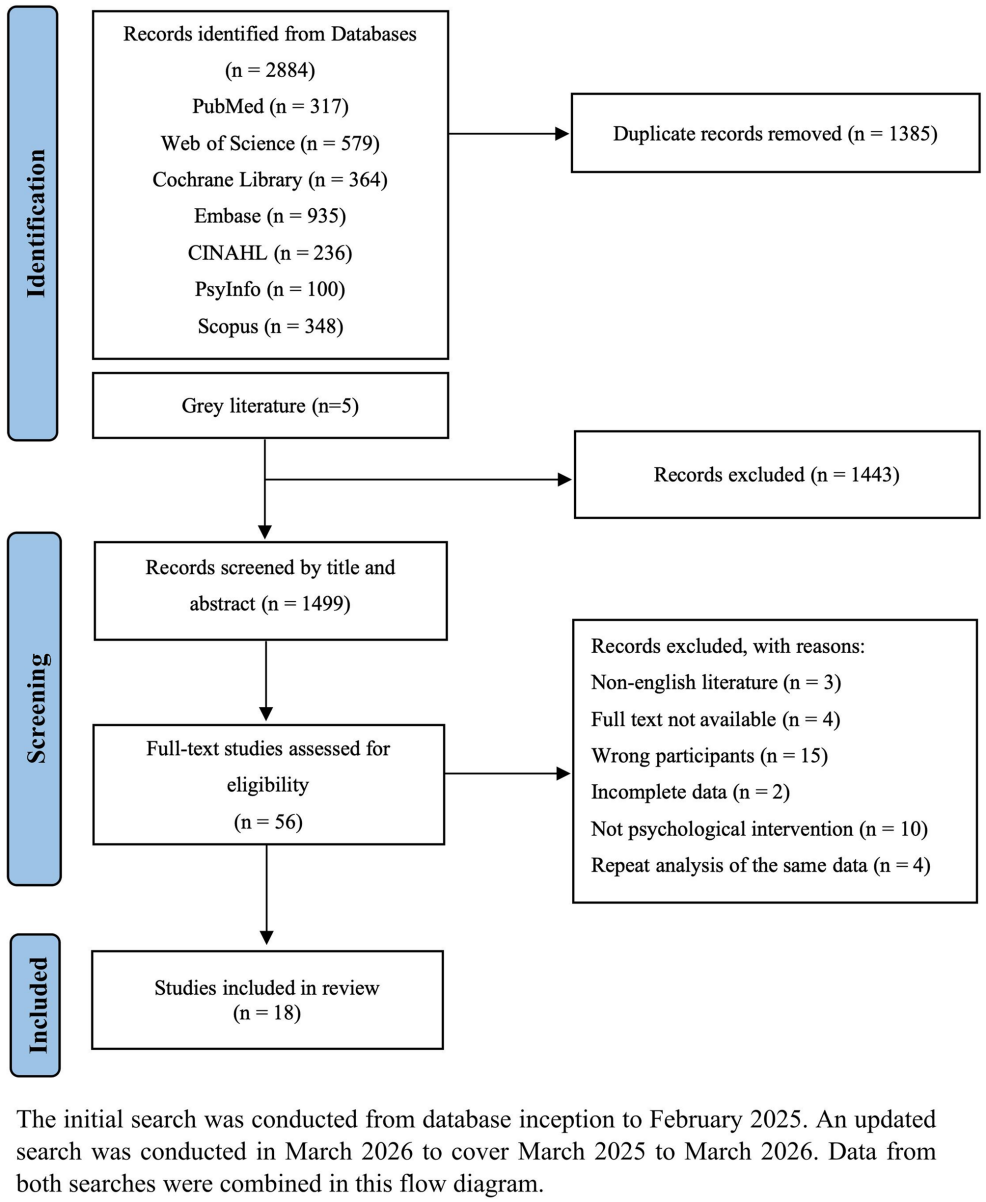
PRISMA flow diagram of study selection

In this study, an initial search of the literature through seven English databases and gray literature yielded 2884 studies. Duplicate studies of 1385 were removed, and the remaining 1499 studies were screened by reading titles and abstracts. After that, 1443 unrelated studies were excluded, and the remaining 56 studies required full-text reading for screening. 18 studies met all inclusion criteria and were included in the synthesis. Consequently, a total of 18 studies were included in the final scoping review. Specific reasons for exclusion were as follows: three studies were not in English, four studies were not available in full text, fifteen studies had the wrong study population, two studies had incomplete data, ten studies had interventions that were not psychological interventions, and four studies analyzed the same dataset repeatedly. The detailed screening process is presented in the PRISMA flowchart (Fig. 1).

### Characteristics of the included studies

#### Geographical characteristics

A total of 18 studies from 10 countries were included in this scoping review, with a geographic distribution covering Asia (China, Korea, Singapore), Europe (Italy, Spain, the Netherlands, Germany), North America (USA, Canada), and Oceania (Australia). In terms of the frequency of country distribution, Spain [47–49], China [50–52], and Germany [53–55] had the highest number of studies (*n* = 3 each, 16.7%), followed by the United States [56, 57] (*n* = 2, 11.1%) and Italy [58, 59] (*n* = 2, 11.1%), and the rest of the countries (Korea, Singapore, Netherlands, Canada, and Australia) had one study each [60–64] (5.6%), which accounted for a relatively low proportion of the studies; Europe contributed the most studies (*n* = 9, 50.0%), followed by Asia (*n* = 5, 27.8%), North America (*n* = 3, 16.7%), and Oceania (*n* = 1, 5.6%).

#### Types of research

Of the 18 studies included, nine were randomized controlled trials (RCTs) [50–52, 56–59, 61, 63], two were mixed-methods study designs [55, 62], five were quasi-experimental designs [47, 53, 54, 60, 64], one was a single-case experimental design [49], and one study was a protocol [48].

#### Age characteristics

The age range of the included studies was focused on 18–65 years, with five studies [48, 52, 55, 58, 61] (*n* = 5, 27.8%) further limiting the upper limit; another four studies [56, 57, 63, 64] (*n* = 4, 22.2%) only set a lower limit of the age of the study population; in addition, one study [59] (*n* = 1, 5.6%) was specifically focused on the older age group of 58–75 years. One study [51] (*n* = 1, 5.6%) was specifically conducted on the adolescent population, while the other two studies [50, 53] (*n* = 2, 11.1%) did not report the age of the participants.

#### Study population

Twelve of the included studies [47–49, 51–57, 60, 61] targeted the MDD population, two studies [58, 59] targeted the BD population, and the remaining four studies [50, 62–64] targeted both the MDD and BD populations.

### Trends of publications

**Fig. 2:**
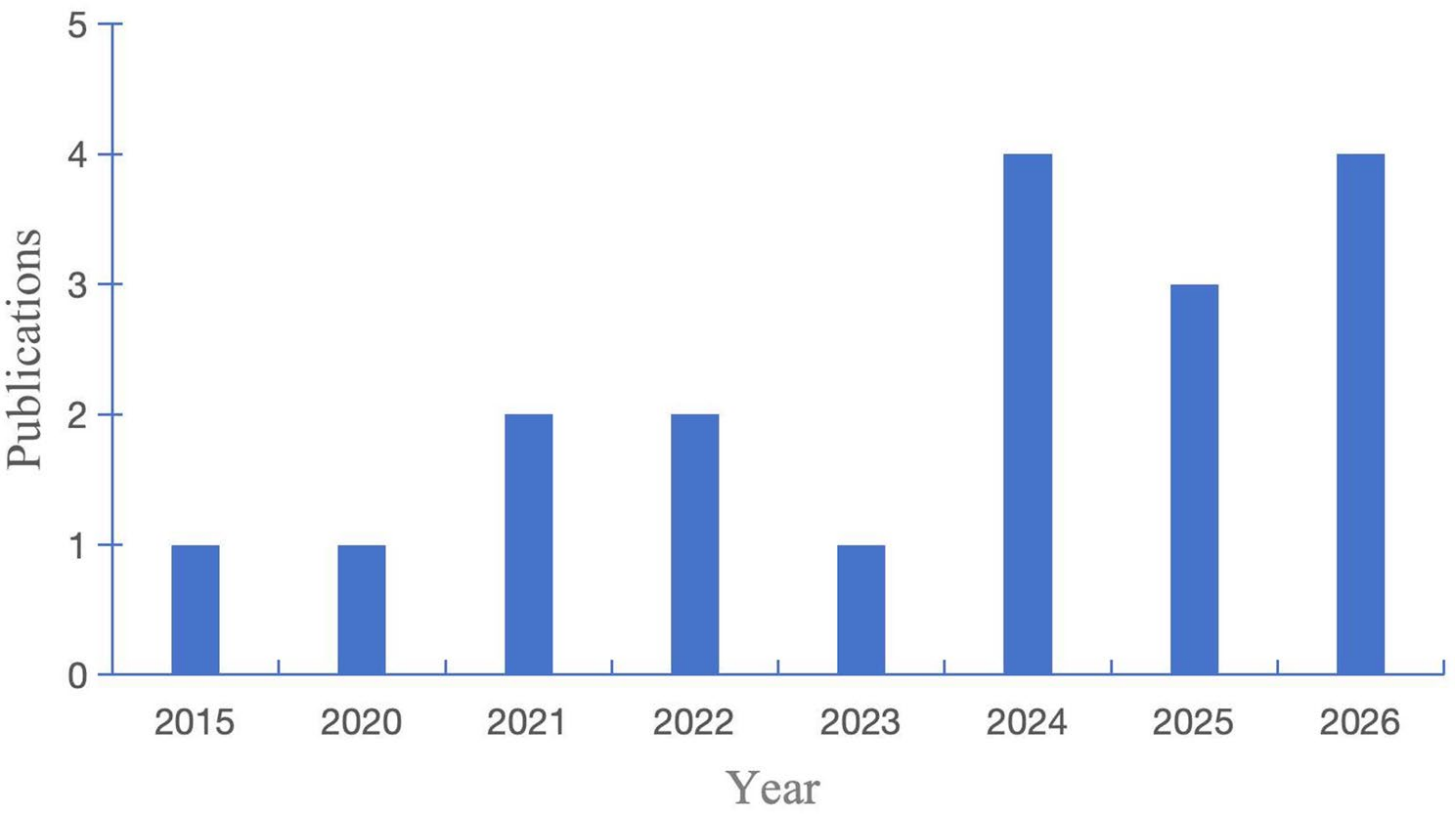
Trends of relevant publications

Most included studies were published after 2020. Only one study was published in 2015. The number of studies showed an increasing trend over time. It peaked at 4 studies in 2024 [55, 57, 59, 62] and 2026 [51, 53, 54, 60] **(**Fig. 2**)**. The data of 2026 only covered the first three months. The full-year numbers may be higher than currently shown.

### Content of VR-based psychological interventions

Interventions in the 18 included studies were all delivered using immersive VR hardware. We conducted an analysis based on the content of interventions. The two main dimensions we focused on were the mode of user engagement within the VR environment and therapeutic content and theoretical orientation (Table 1).

**Table 1:** Types of interventions

Category	Therapeutic Content	User Engagement	Intervention Examples
1. Cognitively Oriented Interventions	Cognitive restructuring, behavioral activation, personal construct theory	Highly interactive (gesture, voice, gaze, therapist communication)	VR-CBT [61], VR-CR [58, 59], VR-BA [49, 56, 57], PCT-VR [48]
2. Experience-Oriented Interventions	Mindfulness, relaxation, EMDR	Non-interactive (passive observation, audio guidance) or mixed	VR-MBI [62], VRelax [63], VR-EMDR [51, 52], VR DE-STRESS [64], virtual reality-based nature immersion [50], virtual reality-based nature embodiment [54], VR-AAI [53]
3. Social Situation Simulation	Social skills training, role-play	Highly interactive (real-time role-play with avatars/therapist)	VR-roleplays [55]
4. Psychoeducational Tools	Psychoeducation, knowledge delivery	Interactive (visualization-based learning)	VRight [47]

One study [60] employed a mixed approach combining social situation simulation (avatar therapy) with cognitive-oriented components (cognitive restructuring and self-compassion training)

#### Types of interventions

##### Interactivity and VR modalities

The level of user interactivity varied across studies. Thirteen studies [47–51, 53–55, 57–61] used interactive interventions, requiring active participation through gestures, voice, gaze, body movements, controller-triggered behaviors, or communication with a therapist. Four studies [52, 56, 62, 64] used non-interactive interventions, where participation involved passive observation or audio-guided immersion. One study [63] adopted a hybrid pattern, incorporating both interactive and non-interactive modules.

##### Therapeutic categories

Interventions were divided into four categories based on their treatment content.

- **Cognitively-oriented interventions.** This category uses VR for interactive exposure and behavioral practice. These interventions use highly interactive scenarios in which users complete structured tasks. Examples include virtual reality-based cognitive behavioral therapy (VR-CBT), virtual reality-based cognitive remediation (VR-CR), virtual reality-based behavioral activation (VR-BA), and virtual reality-based personal construct therapy (PCT-VR).
- **Experience-oriented interventions.** This category uses VR to create immersive states for relaxation and emotional regulation, with a focus on passive or guided immersion. Examples include virtual reality-based mindfulness intervention (VR-MBI), virtual reality-based relaxation training (VRelax), virtual reality eye movement desensitization and reprocessing (VR-EMDR), virtual reality-based stress management program (VR DE-STRESS), virtual reality-based nature immersion, virtual reality-based nature embodiment, and virtual reality-based animal-assisted intervention (VR-AAI).
- **Social situation simulation.** This category uses VR-based social skills training to create highly interactive role-play environments for real-time practice. The primary example is VR roleplays.
- **Psychoeducational tools.** This group uses VR as an interactive learning tool, delivering knowledge through interactive visualization. The example is a virtual reality-based psychoeducational tool (VRight).

One included study [60] applied a mixed approach combining social situation simulation (avatar therapy) with cognitive-oriented components (cognitive restructuring and self-compassion training).

#### Elements of intervention implementation

Elements of VR-based psychological interventions include equipment, period, frequency, and duration. Nine studies [47, 49–52, 55, 60, 61, 64] used a tethered HMD, eight studies [48, 53, 54, 56–59, 62] used a standalone HMD, and only one study [63] used a screenless viewer.

The intervention programs included in the studies showed a high degree of heterogeneity in terms of periodicity, frequency, and duration. Eight studies [49, 51, 52, 54, 56, 57, 61, 63] set 4–12 sessions, with intervention durations focused on 30–50 min. One study [60] extended to 60–90 min per session. And the frequencies mainly 1–2 times per week. Two studies [58, 59] implemented a high-intensity intervention of 24 sessions (2 times per week for 3 months); four studies had ultra-short interventions, including three [47, 53, 62] single-session interventions (6–15 min) and one [54] four-session intervention (approximately 4 min per session); two studies [50, 64] used a short intensive format of 3 sessions delivered once daily (10–60 min per session); one study [55] was an individualized intervention with the therapist deciding the protocol; another study [48] had a more specific protocol: a single high-frequency intervention (10 sessions/day) with booster sessions. Specifics of the intervention implementation refer to Table 2.

**Table 2:** The characteristics of studies (*n* = 18)

Author, Counry, and years	Study design	Sample	Age	Participant	Experimental group			Control group	Outcome measures
					Equipment	Intervention content	cycle/frequency/duration		
Yan, et al. [52] (2025) China	RCT	36	18–45	MDD	Tethered HMD	Centering on eye movement stimulation and role switching, this immersive VR trauma memory reprocessing program (VR-EMDR) employs relaxation and natural imagery (soaring birds, blooming flowers) as guiding elements.	12 sessions over 6 weeks/40–50 min	Waitlist	① Depressive symptoms ② Post-traumatic stress symptoms ⑨ Cognitive function
Perra, et al. [58] (2023) Italy	RCT	39	18–75	BD	Standalone HMD	CBT and Cognitive Rehabilitation-based Immersive VR Cognitive Remediation within a Biopsychosocial Framework. (VR-CR)	24 sessions / two sessions per week over three weeks / 45 min	Waitlist	① Depressive symptoms ⑧ Emotional awareness ⑨ Cognitive function ⑪ Feasibility ⑫ Acceptability ⑬ Satisfaction ⑭ Tolerability ⑮ VR side effects ⑯ Safety
Colombo, et al. [49] (2022) Spain	SCED	8	18–65	MDD	Tethered HMD	A VR-based behavioral activation (VR-BA) intervention program enables patients to complete embodiment training of goal-oriented activities matched with personal values in virtual environments.	4 sessions/ twice a week over two weeks/ 30–45 min	/	① Depressive symptoms ⑦ Emotion regulation ⑩ Daily activity level
Montesano, et al. [48] (2021) Spain	Protocol	75	18–29	MDD	Standalone HMD	By utilizing VR technology, the traditional Personal Construct Therapy (PCT-VR) has been optimized. The program is based on Kelly’s Personal Construct Theory framework, preserving the core four-stage structure of standard PCT (Role Construct Repertory Test, Construct Exploration, Cognitive Restructuring, and Behavioral Experiment). During the 3rd session (Construct Visualization) and the 10th session (Consolidation), it achieves three-dimensional immersive presentation and interactive exploration of personal constructs.	a maximum of 10 individual 1-h sessions, with one or two booster sessions at about 3 months after the tenth session.	CBT/PCT	① Depressive symptoms ③ Anxiety symptoms ④ Symptoms of stress ⑤ General psychological ⑬ Satisfaction ⑰ Treatment compliance
Lee, et al. [61] (2025) Korea	RCT	19	19–50	MDD	Tethered HMD	Developing a VR-based CBT treatment program, including virtual therapists, training in mindfulness and attachment, emotional self-regulation, social interaction skills, and mindfulness-based exercises (VR-CBT)	6 sessions / once a week for 6 weeks/15–30 min	TAU	① Depressive symptoms ⑥ Suicidal ideation ⑦ Emotion regulation
Paul, et al. [57] (2024) USA	RCT	13	≥ 18	MDD	Standalone HMD	Developed a VR system-based augmented reality behavioral activation therapy (VR-BA), which achieves innovative digital intervention transformation while preserving the core elements of traditional behavioral activation therapy through expert-guided immersive activity protocols [Although this protocol adopts XR terminology, its actual implementation is entirely based on the virtual reality technology framework (without involving augmented/mixed reality components)].	4 sessions/once per week/30–50 min	Traditional BA	① Depressive symptoms ⑪ Feasibility ⑫ Acceptability ⑭ Tolerability ⑮ VR side effects
Blackmore, et al. [62] (2024) Australia	Mixed-methods	19	18–65	MDD + BD	Standalone HMD	The VR-based mindfulness intervention program includes anchoring attention through natural imagery guidance, cyclic exercises focusing on bodily sensations-breathing-environmental details, and embedded voice-guided training (VR-MBI).	1 session/ 15 min	/	③ Anxiety symptoms ⑦ Emotion regulation Qualitative data
Migoya-Borja, et al. [47] (2020) Spain	Quasi-experiment	13	18–65	MDD	Tethered HMD	A VR-based psychological education tool that simulates one-on-one interaction scenarios, with dialogue topics centered on depression symptom recognition, featuring virtual characters matching the user’s gender (VRight).	1 session/ 10 min	/	⑧ Emotional awareness ⑫ Acceptability ⑬ Satisfaction
Paul, et al. [56] (2022) USA	RCT	5	≥ 18	MDD	Standalone HMD	VR-based Behavioral Activation Therapy (VR-BA) sets goals through mood-activity logs, simulates real-world activities using passive 360° videos, and reinforces intervention effects through regular review of logs and activity schedules.	4 sessions/ once per week for 3 weeks/ 50 min	Traditional BA/TAU	① Depressive symptoms ⑦ Emotion regulation ⑪ Feasibility ⑭ Tolerability ⑮ VR side effects ⑯ Safety
Primavera, et al. [59] (2024) Italy	RCT	15	58–75	BD	Standalone HMD	CBT and Cognitive Rehabilitation-based Immersive VR Cognitive Remediation within a Biopsychosocial Framework, (VR-CR)	24 sessions / two sessions per week over three months/45 min	TAU	① Depressive symptoms ⑨ Cognitive function
Veling, et al. [63] (2021) Netherlands	RCT	29	> 18	MDD + BD	Screenless Viewer	VR-based relaxation therapy (VRelax) constructs an immersive VR environment through 360° nature videos (beaches, coral reefs, the Alps, etc.), allowing users to freely explore scenes and trigger meditation guidance, muscle relaxation audio, or interactive games (such as bubble bursting and melody generation) by gazing at hotspots, achieving multi-sensory relaxation.	10 sessions/ once a day/10 min	Standard relaxation exercises	③ Anxiety symptoms ④ Symptoms of stress ⑦ Emotion regulation ⑮ VR side effects
Holsteg, et al. [55] (2024) Germany	Mixed-methods	8	19–58	MDD	Tethered HMD	VR-based role play, with flexible adjustment of difficulty, is a personalized treatment for patients. (two scenarios: boss roleplay or colleague roleplay from the patient’s perspective)	decided by therapists	/	⑫ Acceptability ⑬ Satisfaction ⑯ Safety Qualitative data
Shah, et al. [64] (2015) Singapore	Quasi-experiment	22	21–65	MDD + BD	Tethered HMD	VR-based stress-reduction intervention program, with core components comprising face-to-face psychoeducation and immersive relaxation training, equipped with a step-by-step practice manual and home consolidation audio (VR DE-STRESS).	3 sessions/ once a day/1-hour	/	① Depressive symptoms ③ Anxiety symptoms ④ Symptoms of stress ⑱ Relaxation levels ⑲ knowledge level Participant Feedback
Gao, et al. [50] (2025) China	RCT	60	NR	MDD + BD	Tethered HMD	A VR-based environmental immersion intervention employing commercially available nature and urban scenarios (Nature Treks; Dafen Oil Painting Village) to induce fascination-driven positive distraction through task-free exploratory experiences, with audiovisual integration.	3 sessions/ once a day/10 min	Virtual city immersion	① Depressive symptoms, ③ Anxiety symptoms ④ Symptoms of stress ⑳ Psychosocial Functioning Physiological measure
Giguère, et al. [60] (2026) Canada	Quasi-experimental Study	12	≥ 18	MDD	Tethered HMD	VR-based Avatar Therapy immerses participants in personalized emotional contexts through therapist-embodied avatars of significant others, enables experiential processing of depressive symptoms and interpersonal conflicts via real-time role-playing, and integrates behavioral activation, cognitive restructuring, and self-compassion training.	at least eight weekly sessions /60–90 min	/	① Depressive symptoms, ③ Anxiety symptoms ⑫ Acceptability ⑳ Psychosocial Functioning Response rate
Yan, et al. [51] (2026) China	RCT	54	13–18	MDD	Tethered HMD	VR-based Eye Movement Desensitization and Reprocessing (VR-EMDR), tracks eye movements through built-in HMD sensors, and guides participants through five structured phases: relaxation, memory evaluation with self-compassion, guided and independent reprocessing, and body scan.	12 sessions/6 weeks/40–50 min	Waitlist	① Depressive symptoms; ⑨ Cognitive function physiological measure
Lütt, et al. [54] (2026) Germany	Quasi-experimental study	20	18–65	MDD	Standalone HMD	VR-based nature embodiment intervention enables patients to embody a growing rainforest tree from seed to maturity through synchronous body movements (e.g., arms corresponding to branches), eliciting virtual body ownership and nature connectedness.	4 sessions/once a week/4 minutes 20 s	Healthy controls	⑯ Safety ⑳ Psychosocial Functioning Symptom burden physiological measure
Willms, et al. [53] (2026) Germany	Quasi-experimental study	33	NR	MDD	Standalone HMD	VR-based animal-assisted intervention (VR-AAI) enables participants to interact with a lifelike virtual therapy dog or VR-based fantastical creature through controller-triggered behaviors (approaching, sitting, lying down, playful movements, physical-contact animations, play/retrieval sequences), following a semi-structured, self-directed logic	Single session/6 minutes	/	① Depressive symptoms ⑮ VR side effects psychophysiological measure

Notes. NR, not report; SCED, single-case experimental design; HMD, head-mounted display; CBT, cognitive behavior therapy; PCT, personal construct therapy; TAU, treatment as usual; BA, behavioral activation therapy. ① Depressive symptoms; ② Post-traumatic stress symptoms; ③ Anxiety symptoms; ④ Symptoms of stress; ⑤ General psychological distress; ⑥ Suicidal ideation; ⑦ Emotion regulation; ⑧ Emotional awareness; ⑨ Cognitive function; ⑩ Daily activity level; ⑪ Feasibility activity level; ⑪ Feasibility; ⑫ Acceptability; ⑬ Satisfaction; ⑭ Tolerability; ⑮ VR side effects; ⑯ Safety; ⑰ Treatment compliance; ⑱ Relaxation levels; ⑲ knowledge level; ⑳ Psychosocial Functioning

### Feasibility and efficacy assessments

The outcome measures primarily focused on feasibility and efficacy-related assessments. Ten studies [47, 48, 53–58, 60, 63] collected indicators related to feasibility. Findings included reports of participant acceptability [47, 55, 57, 58], satisfaction [47, 55, 58], and tolerability [56–58]. Four studies [54–56, 58] described the safety of the VR systems, with one [55] noting patient discomfort during role-play scenarios and another [54] reporting VR simulator sickness. Four studies [56–58, 63] reported side effects such as nausea and dizziness. One study [63] reported that 14.3% of participants stopped using VR due to motion sickness. However, Willms et al. [53] reported that there was no adverse events occurred during the intervention.

Efficacy-related outcomes were grouped into six types.

1. **Depressive and Anxiety Symptoms.** Thirteen studies [47, 49–53, 56–61, 64] reported reductions in scores for depressive symptoms. Five studies [50, 62–64] showed reductions in scores for anxiety symptoms.
2. **Cognitive Function.** Four studies [51, 52, 58, 59] reported improvements in scores on cognitive tests (e.g., memory, attention). One study [59] noted that the degree of improvement differed between younger and older patients.
3. **Emotion Regulation.** Eight studies reported outcomes related to emotion regulation. Six studies [49, 50, 56, 61–63] reported reductions in negative emotional states or increases in positive mood. Enhanced scores for emotion perception were observed in one study [58], and increased levels of perceived relaxation in another [64].
4. **Activity**,** Rhythms**,** and Knowledge.** One study [49] reported an increase in time spent on daily activities. Another study [58] presented improved scores for biological rhythm. An additional study mentioned an increase in participants’ knowledge levels.
5. **Stress Perception.** Three studies assessed stress. One study [63] reported reductions in perceived stress scores. Two studies observed decreases in both objective and subjective stress levels [50, 64].
6. **Psychosocial Functioning.** One study [54] reported the improvements in compassion and reductions in individual symptom burden. One study [60] observed the improvements in self-esteem. Another study [60] reported improvements in quality of life, and functioning.

One registered protocol [48] listed its intended outcomes, including depressive symptoms, anxiety, stress, distress, adherence, and satisfaction, but it did not present the corresponding results. Another study collected participant feedback by evaluation scales. The reported feedback covered perceived benefits, preferred intervention components, and suggestions for improvement [64]. Four studies included physiological measures as outcomes. Gao et al. [50] measured heart rate as an objective stress indicator. Yan et al. [51] reported changes in small-world network attributes. Lütt et al. [54] recorded electrodermal activity. Willms et al. [53] measured oxytocin levels.

### Qualitative integration of interventions

Two studies used qualitative interviews to examine user experiences in VR-based psychological interventions. Holsteg et al. [55] focused on interactional challenges in VR roleplays, while Blackmore et al. [62] explored broader perceptions of VR-MBI, including engagement, accessibility, and perceived therapeutic utility.

#### Interaction design

In VR roleplays, interaction design barriers were related to self-representation, agent behavior, and conversational flow. Participants reported that invisible hands and feet weakened their sense of embodiment. They also noted that virtual agents exhibited delayed or unnatural turn-taking, which disrupted dialogue rhythm and created uncertainty about response timing. Some interjections were perceived as interruptions. In addition, co-worker agents failed to draw on shared knowledge from prior interactions, reducing interactional coherence [55].

In contrast, participants in the VR-MBI valued visual immersion. Many reported that it helped them engage more quickly in the task and reduce attentional fatigue. However, some participants identified a conflict. They were instructed to close their eyes within a visually detailed virtual forest environment. This mismatch between instructions and visual stimuli led to confusion [62].

#### Environmental fidelity

Participants’ perceptions of environmental fidelity differed significantly between technical performance and experiential function. In the VR-roleplays, participants criticized the virtual environment for blurry visuals, unrealistic details, low audio quality, and limited contextual realism. These factors jointly reduced their sense of presence [55].

In the VR-MBI study, participants mainly evaluated environmental features based on functional outcomes. Many found the virtual environment helpful for reducing external distractions and forming a private “bubble” via headphone-induced sensory isolation. However, some participants reported that they were distracted by the visual content. This may indicate that environmental fidelity interacts with individual attention characteristics and task requirements [62].

#### Hardware suitability

The VR-roleplays study identified multiple hardware-related barriers. Participants reported physical discomfort from headset weight, required time to adapt to the visual display, and expressed dissatisfaction with controller-based interactions [55].

Despite these limitations, participants in the mindfulness intervention reported therapeutic benefits. They described improved emotional regulation, greater ability to disengage from distressing thoughts, and increased motivation to counter depression-related lethargy. These findings suggest that the perceived psychological benefits of virtual environments may offset hardware-related discomfort for many users [62].

#### User experience

User experiences differed significantly across intervention types. In the VR-roleplays condition, participants reported persistent difficulties with situational immersion, unnatural actions, non-adaptive scenarios, and ambiguous negotiation outcomes [55].

However, participants in the VR-MBI felt psychologically safe in the VR environment. They considered it less threatening than real-world settings. Many considered it a proactive early intervention tool, especially for interrupting repetitive negative thinking before it escalated [62]. This comparison showed that the different VR‑based therapeutic approaches may result in distinct user experiences.

#### Reality transfer capability

VR-Roleplays participants expressed the uncertainty about how to “act properly” in such an environment and felt their responses lacked authenticity, raising concerns about the transferability of skills acquired in simulation [55]. On the other hand, VR-MBI participants thought the technology helpful for guiding beginners. They reported that external cues helped them ease into mindfulness practice when struggling with body awareness [62].

## Discussion

### Summary of principal findings

This scoping review identified 18 studies on immersive VR-based psychological interventions for MDD and BD. One study was published in 2015, the remaining 17 were published between 2020 and 2026, rapidly expanding over the past few years. This trend is consistent with the growth of VR research in mental health, as documented in previous systematic reviews [65]. However, the evidence base for mood disorders is less extensive than that for anxiety disorders and PTSD [42, 44, 66, 67]. Controlled trials of VR exposure therapy for anxiety emerged in the early 2000s, with meta-analyses available before 2010 [68–70]. In contrast, methodologically rigorous trials for mood disorders have only begun to accumulate since approximately 2020 [66]. Recent systematic reviews continue to describe the evidence base as preliminary and of low to moderate quality [71].

The intervention designs across the 18 studies varied significantly. We organized these interventions into four categories: cognitively oriented, experience-oriented, social situation simulation, and psychoeducational tools. According to the analysis of content, cognitive and experience-oriented interventions were primarily based on two well-established therapeutic approaches. Seven studies used cognitive behavioral therapy (CBT) and its core components, including behavioral activation and cognitive remediation [48, 49, 56–59, 61]. Mindfulness and relaxation training served as the core component in five studies [51, 52, 62–64]. Two studies were social simulation and psychoeducation [47, 55]. Three studies applied emerging VR-assisted psychological interventions, including virtual nature immersion [50], virtual embodiment as a tree [54], and animal-assisted intervention [53]. The remaining one study [60] applied a mixed intervention pattern of cognitive-oriented components and scenario simulation. This distribution is consistent with previous studies. CBT is the most commonly used and empirically supported approach in digital mental health interventions. In the analysis of digital health applications for depression available in Germany, CBT was identified as the dominant therapeutic modality in mental health [72]. A systematic review by Wang et al. reported that VR-based CBT has shown promising effects across various mental health conditions [73]. Another study found that virtual reality exposure-based Cognitive Behavioral Therapy (VRE-CBT) has great potential for treating anxiety disorders, but more high-quality evidence is needed [74]. Recent years have seen an increase in VR-based mindfulness and relaxation interventions. Similarly, emerging VR approaches, including virtual nature immersion, embodiment (e.g., embodying a tree), and animal-assisted interventions, have been reported to improve mood, reduce stress, and enhance nature connectedness [50, 53, 54]. These findings from the included studies are consistent with present reports [75–77]. Compared with CBT-based or mindfulness-based VR interventions, these emerging approaches have been less frequently examined. Existing evidence is mainly based on small sample or preliminary studies. However, despite the favorable results observed in these studies, none have been incorporated into mainstream clinical practice. More evidence from rigorous studies is needed to establish their efficacy. Further research with larger sample sizes and rigorous designs is needed [78].

In addition to intervention characteristics, this review summarized the reported outcomes from the included studies. The majority of studies reported improvements in depressive and anxiety symptoms, as well as positive feasibility outcomes like acceptability and satisfaction. However, side effects such as nausea and dizziness were reported in four studies, and one registered protocol did not produce results.

The analysis of the included studies revealed significant heterogeneity in intervention design and implementation. Interactivity ranged from passive observation to active participation involving gestures, voice, gaze, body movements or controller-triggered behaviors. The duration ranged from a single 4-minute session to an extensive 24-session program conducted over a period of three months [47–64]. This methodological variability restricts the integration of evidence and presents difficulties for cross-study comparisons in the absence of formalized frameworks for intervention or established reporting standards [79, 80]. The qualitative synthesis of this scoping review identified interaction design and environmental fidelity as factors influencing users’ sense of presence and participation in the included studies. This finding is consistent with prior research on digital interventions. Design features such as interface aesthetics, interactivity, and personalization have been shown to affect user engagement and subjective experience [81–84]. The large heterogeneity across interventions observed in this review highlights the need for standardized reporting frameworks and implementation guidelines, as previously suggested [85].

This review revealed several research gaps in the included studies. Although adolescence is a period with a high incidence of mood disorders, only one study [51] focused on adolescent populations. Most research was centered on adults aged 18–65 years [47–49, 52, 55–58, 61–64], with only one study specifically examining older adults aged 58–75 years [59]. Two studies did not specify the age of the participants [50, 53]. Of the 18 included studies, twelve (66.7%) were conducted in Western countries. This may limit the generalizability of findings across diverse cultural contexts. Interventions were mainly implemented in hospital or laboratory settings, and none were conducted in community or home environments. Heterogeneity was also observed in outcome measurement. Assessment instruments and evaluation time points varied across studies. Moreover, most included studies provided only limited information about device-related side effects. Only two studies [55, 62] used qualitative interviews to explore participants’ VR user experience in detail. Also, few studies examined the mechanisms through which VR interventions may produce therapeutic effects on mood disorders, with the exception of Willms et al. [53], who measured oxytocin levels as a potential neurobiological correlate. Future research should expand age coverage to include adolescent and older adult populations. Studies in underrepresented geographic regions and community settings are also needed. Standardized outcome measurement protocols would improve cross-study comparability. And standardized frameworks should also be developed to guide protocol design. Systematic assessment of adverse effects and user experience is warranted. Research on intervention mechanisms, if feasible, would help clarify the effectiveness of VR interventions.

### Limitation

There are several limitations that need to be acknowledged. First, only studies published in English were included, which may lead to language bias. Second, the inclusion of study protocols broadened the scope of intervention mapping, potentially omitting relevant findings from completed trials. Third, this review only focuses on head-mounted display-based VR interventions, which may limit the breadth of included evidence. Finally, although the updated search extended to March 2026, the rapid evolution of VR technology means that newer devices or intervention approaches may have emerged after the search date. These limitations suggest that future reviews should consider non-English literature, prioritize completed trials, and explore VR psychological interventions beyond head-mounted display systems.

## Conclusions

This scoping review provides a comprehensive synthesis of the application of VR-based psychological interventions for MDD and BD. A total of 18 studies were included in this review. Study designs included randomized controlled trials, single-case experimental designs, quasi-experimental designs, mixed-methods studies, and one protocol. The findings reveal that research in this area is still developing, with considerable heterogeneity across study design, outcome measurement, and reporting practices. To confirm the efficacy in MDD and BD, future studies should focus on developing standardized intervention frameworks, conducting rigorous trials with larger sample sizes, and covering a wider age range of people.

## Electronic supplementary material

Below is the link to the electronic supplementary material.

Supplementary Material 1

## Data Availability

The data extraction table supporting this scoping review is available in the Supplementary Material. All original data were obtained from published studies cited in the reference list.

## Abbreviations

MD: Mood disorders
VR: Virtual reality
MDD: Major depressive disorder
BD: Bipolar disorder
SCED: Single-case experimental design
RCT: Randomized controlled trial
HMD: Head-mounted display
CBT: Cognitive behavior therapy
PCT: Personal construct therapy
TAU: Treatment as usual
BA: Behavioral activation therapy
EMDR: Eye movement desensitization and reprocessing
CR: Cognitive rehabilitation
MBI: Mindfulness-based intervention
