# Supplementary material for: Virtual reality in psychological interventions for mood disorders: a scoping review
Source: BMC Psychiatry. 2026 Apr 21;26:441. doi: 10.1186/s12888-026-08088-9 (PMC13231538; doi:10.1186/s12888-026-08088-9)
Supplement: Supplementary file 1 — Supplementary Material 1 [file 12888_2026_8088_MOESM1_ESM.docx]

**Search strategy**.

**1. Search strategy for potential eligible studies:**

1. **Search strategy via PubMed**

| **Number** | **Search terms** |
| --- | --- |
| #1 | (("mood disorders"[MeSH Terms] OR "mood disorder"[Title/Abstract] OR "affective disorder*"[Title/Abstract] OR "bipolar disorder*"[Title/Abstract] OR "bipolar mood disorder*"[Title/Abstract] OR "manic depression"[Title/Abstract] OR "depressive disorder*"[Title/Abstract] OR "depressive syndrome*"[Title/Abstract] OR "major depressive disorder"[Title/Abstract] OR "depression"[MeSH Terms] OR "depression"[Title/Abstract])) |
| #2 | (("virtual reality"[MeSH Terms] OR "virtual reality"[Title/Abstract] OR "VR"[Title/Abstract] OR "VR-based"[Title/Abstract] OR "immersive technology"[Title/Abstract] OR "head mounted display"[Title/Abstract] OR "HMD"[Title/Abstract])) |
| #3 | (("psychotherapy"[MeSH Terms] OR "psychotherapy"[Title/Abstract] OR "psychotherapies"[Title/Abstract] OR "psychological therapy"[Title/Abstract] OR "psychological therapies"[Title/Abstract] OR "psychological intervention*"[Title/Abstract])) |
| #4 | #1 AND #2 AND #3 |

1. **Search strategy for EMBASE**

| **Number** | **Search terms** |
| --- | --- |
| #1 | ('mood disorder':ab,ti OR 'affective disorder*':ab,ti OR 'bipolar disorder*':ab,ti OR 'bipolar mood disorder*':ab,ti OR 'manic depression':ab,ti OR 'depressive disorder*':ab,ti OR 'depressive syndrome*':ab,ti OR 'major depressive disorder':ab,ti OR 'depression':ab,ti) |
| #2 | 'mood disorder'/exp |
| #3 | ('virtual reality':ab,ti OR 'virtual realities':ab,ti OR 'educational virtual reality':ab,ti OR 'educational virtual realities':ab,ti OR 'instructional virtual reality':ab,ti OR 'instructional virtual realities':ab,ti OR 'VR':ab,ti OR 'VR-based':ab,ti OR 'immersive technology':ab,ti OR 'head-mounted display':ab,ti OR 'HMD':ab,ti) |
| #4 | ('psychotherapy':ab,ti OR 'psychotherapies':ab,ti OR 'psychological therapy':ab,ti OR 'psychological therapies':ab,ti OR 'psychological intervention*':ab,ti) |
| #5 | #1 OR #2 |
| #6 | #3 AND #4 AND #5 |

1. **Search strategy for Web of Science**

| **Number** | **Search terms** |
| --- | --- |
| #1 | TS=(''mood disorders'' OR ''mood Disorder'' OR ''Affective Disorders'' OR ''Affective Disorder'' OR ''Bipolar Disorders'' OR ''Bipolar Disorder'' OR ''Bipolar Mood Disorder'' OR ''Bipolar Mood Disorders'' OR ''Manic Depression'' OR ''Depressive Disorders'' OR ''Depressive Disorder'' OR ''Depressive Syndrome'' OR ''Depressive Syndromes'' OR ''major depressive disorder'' OR ''depression'') |
| #2 | TS=(''Virtual Reality'' OR ''Virtual Realities'' OR ''Educational Virtual Realities'' OR ''Educational Virtual Reality'' OR ''Instructional Virtual Realities'' OR ''Instructional Virtual Reality'' OR ''VR'' OR ''VR-based'' OR ''immersive technology'' OR ''head-mounted display'') |
| #3 | TS=(''psychotherapy'' OR ''psychotherapies'' OR ''psychological therapy'' OR ''psychological therapies'' OR ''psychological intervention*'' ) |
| #4 | #1 AND #2 AND #3 |

1. **Search strategy for Global Health, Cumulative Index to Nursing and Allied Health Literature (CINAHL), PsycINFO**

| **Number** | **Search terms** |
| --- | --- |
| #1 | TI=(''mood disorders'' OR ''mood Disorder'' OR ''Affective Disorders'' OR ''Affective Disorder'' OR ''Bipolar Disorders'' OR ''Bipolar Disorder'' OR ''Bipolar Mood Disorder'' OR ''Bipolar Mood Disorders'' OR ''Manic Depression'' OR ''Depressive Disorders'' OR ''Depressive Disorder'' OR ''Depressive Syndrome'' OR ''Depressive Syndromes'' OR ''major depressive disorder'' OR ''depression'') OR AB=(''mood disorders'' OR ''mood Disorder'' OR ''Affective Disorders'' OR ''Affective Disorder'' OR ''Bipolar Disorders'' OR ''Bipolar Disorder'' OR ''Bipolar Mood Disorder'' OR ''Bipolar Mood Disorders'' OR ''Manic Depression'' OR ''Depressive Disorders'' OR ''Depressive Disorder'' OR ''Depressive Syndrome'' OR ''Depressive Syndromes'' OR ''major depressive disorder'' OR ''depression'') |
| #2 | TI =(''Virtual Reality'' OR ''Virtual Realities'' OR ''Educational Virtual Realities'' OR ''Educational Virtual Reality'' OR ''Instructional Virtual Realities'' OR ''Instructional Virtual Reality'' OR ''VR'' OR ''VR-based'' OR ''immersive technology'' OR ''head-mounted display'') OR AB=(''Virtual Reality'' OR ''Virtual Realities'' OR ''Educational Virtual Realities'' OR ''Educational Virtual Reality'' OR ''Instructional Virtual Realities'' OR ''Instructional Virtual Reality'' OR ''VR'' OR ''VR-based'' OR ''immersive technology'' OR ''head-mounted display'') |
| #3 | TI=(''psychotherapy'' OR ''psychotherapies'' OR ''psychological therapy'' OR ''psychological therapies'' OR ''psychological intervention*'' ) OR AB=(''psychotherapy'' OR ''psychotherapies'' OR ''psychological therapy'' OR ''psychological therapies'' OR ''psychological intervention*'' ) |
| #4 | #1 AND #2 AND #3 |

1. **Search strategy for Cochrane library**

| **Number** | **Search terms** |
| --- | --- |
| #1 | MeSH descriptor: [Mood Disorders] explode all trees |
| #2 | MeSH descriptor: [Bipolar disorder] explode all trees |
| #3 | MeSH descriptor: [Depression] explode all trees |
| #4 | (''Affective Disorders'' OR ''Affective Disorder'' OR ''Bipolar Disorders'' OR ''Bipolar Disorder'' OR ''Bipolar Mood Disorder'' OR ''Bipolar Mood Disorders'' OR ''Manic Depression'' OR ''Depressive Disorders'' OR ''Depressive Disorder'' OR ''Depressive Syndrome'' OR ''Depressive Syndromes'' OR ''major depressive disorder'' OR ''depression''):ti,ab,kw |
| #5 | (''Virtual Reality'' OR ''Virtual Realities'' OR ''Educational Virtual Realities'' OR ''Educational Virtual Reality'' OR ''Instructional Virtual Realities'' OR ''Instructional Virtual Reality'' OR ''VR'' OR ''VR-based'' OR ''immersive technology'' OR ''head-mounted display''):ti,ab,kw |
| #6 | (''psychotherapy'' OR ''psychotherapies'' OR ''psychological therapy'' OR ''psychological therapies'' OR ''psychological intervention*'' ) :ti,ab,kw |
| #7 | #1 OR #2 OR #3 OR #4 |
| #8 | #5 AND #6 AND #7 |

1. **Search strategy for Scopus**

| **Number** | **Search terms** |
| --- | --- |
| #1 | (TITLE-ABS-KEY("mood disorder*" OR "affective disorder*" OR "bipolar disorder*" OR "bipolar mood disorder*" OR "manic depression" OR "depressive disorder*" OR "depressive syndrome*" OR "major depressive disorder" OR "depression") |
| #2 | (TITLE-ABS-KEY("virtual reality" OR "virtual realities" OR "educational virtual reality" OR "educational virtual realities" OR "instructional virtual reality" OR "instructional virtual realities" OR "VR" OR "VR-based" OR "immersive technology" OR "head-mounted display" OR "HMD") |
| #3 | (TITLE-ABS-KEY("psychotherapy" OR "psychotherapies" OR "psychological therapy" OR "psychological therapies" OR "psychological intervention*") |
| #4 | #1 AND #2 AND #3 |
